# Supplementary material for: Chromothripsis during telomere crisis is independent of NHEJ, and consistent with a replicative origin
Source: Genome Res. 2019 May;29(5):737–49. doi: 10.1101/gr.240705.118 (PMC6499312; doi:10.1101/gr.240705.118)
Supplement: Supplemental Material [file supp_gr.240705.118_Supplemental_file_1.zip › contigs/annotated_contigs/DB116/contig.2.DB116_length_697_mean_cov_5.38020086083.docx]

**DB116_length_697_mean_cov_5.38020086083**

```
TTACTGGCATTCCAGGAACCACTGGGGTATGAAAAAAAACTCTTGCAGCTAGCTTGGTGTCTGCCCAAACAGCCGCCCAGTTTTGTGCT
 >chr2:172618062-172618416 + E=2e-196
TGAAACCCAGGGCCCTGGTGGTGTAGGCACCGGAAGGAATCTCCTGGTCTGCAGGTTGTGAAGACCGTGGGAAAAGTGCAGTACCTGGG

CCGGAGTGCACTGTTCCTCACAGTACAGTCCCTAATGGCTTTCCTTGGCTAGGAGAGGGAGTTCCCTGACCCCTTGTGCTTCCTGGGTA

AGCAACGCCCCACCCTGCTTCGGCTCGCCCTTGTGGGCTGCACCCACTGTCCAACCAGTCCCAGTGAGATGAACCAGGTACCT|CAG|C
                                                                                    >ch
CTTGCAAATAGCTGCCCCTTGGACACCTTTTCAACCAGGGTAGGTTTGGGCAAAAGATAGATAGGCCTTGTGAAAGCCCTGGAAGCTGA
r2:172618995-172619342 + E=5e-197
GCAGTTTAGGCAGGAAAATCAGGGTTTCAGGTACCTTGAACGTGGTCGAGAAGAGAATGAAGTGAGTTCCAGGTAGGCATAACCCACTG

GCCCAGCAGACTTTTTGCCCCCTGGGAAAGAACACAACTACAGGAGGGCCTCTAAAATGAGGCACCCGAGGGCAGTGCTGGGCCTGTCT

TTTCTCCTAAAGTCAACTGGGTCACTTTTTAAAAGATAATTCTGTTGTCCCTAATATGTTATTTTTTCAATGCAAA
```
